# Relationship between the binding free energy and PCBs’ migration, persistence, toxicity and bioaccumulation using a combination of the molecular docking method and 3D-QSAR

**DOI:** 10.1186/s13065-018-0389-2

**Published:** 2018-02-23

**Authors:** Xiao-Hui Zhao, Xiao-Lei Wang, Yu Li

**Affiliations:** 1 College of Environmental Science and Engineering, North China Electric Power University, No. 2, Beinong Road, Beijing, 102206 China; 2 The Moe Key Laboratory of Resources and Evironmental Systems Optimization, North China Electric Power University, Beijing, 102206 China

**Keywords:** Polychlorinated biphenyl, Molecular docking, Biphenyl dioxygenase, Pearson correlation, Three-dimensional quantitative structure–activity relationship

## Abstract

The molecular docking method was used to calculate the binding free energies between biphenyl dioxygenase and 209 polychlorinated biphenyl (PCB) congeners. The relationships between the calculated binding free energies and migration (octanol–air partition coefficients, *K*_OA_), persistence (half-life, *t*_1/2_), toxicity (half maximal inhibitory concentration, IC_50_), and bioaccumulation (bioconcentration factor, BCF) values for the PCBs were used to gain insight into the degradation of PCBs in the presence of biphenyl dioxygenase. The relationships between the calculated binding free energies and the molecular weights, *K*_OA_, BCF, and *t*_1/2_ values for the PCBs were statistically significant (*P* < 0.01), whereas the relationship between the calculated binding free energies and the IC_50_ for the PCBs was not statistically significant (*P* > 0.05). The electrostatic field, derived from three-dimensional quantitative structure–activity relationship studies, was a primary factor governing the binding free energy, which agreed with literature findings for *K*_OA_, *t*_1/2_, and BCF. Comparative molecular field analysis and comparative molecular similarity indices analysis contour maps showed that the binding free energies, *K*_OA_, *t*_1/2_, and BCF values for the PCBs decreased simultaneously when substituents with electropositive groups at the 3-position or electronegative groups at the 3′-position were introduced. This indicated the binding free energy was correlated with the persistent organic pollutant characteristics of PCBs. Furthermore, low binding free energies improved the degradation of the PCBs and simultaneously decreased the *K*_OA_, *t*_1/2_, and BCF values, thereby reducing the persistent organic pollutant characteristics of PCBs in the environment. These results are expected to be beneficial in providing a theoretical foundation for further elucidation of the degradation and molecular modification of PCBs.

**Electronic supplementary material:**

The online version of this article (10.1186/s13065-018-0389-2) contains supplementary material, which is available to authorized users.

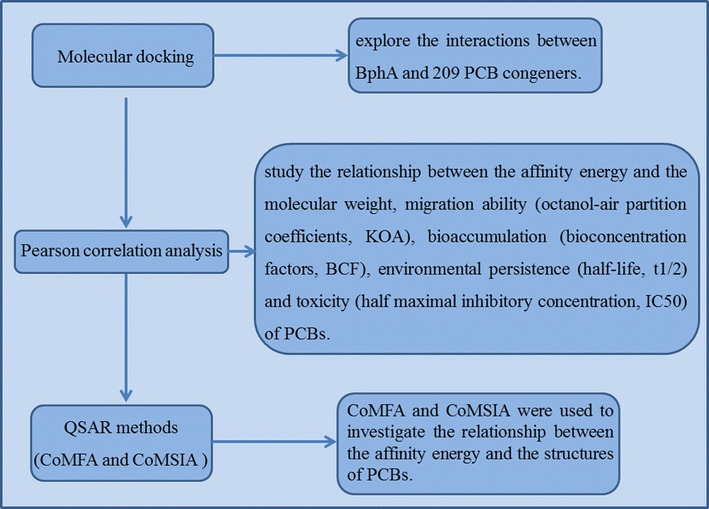

## Introduction

Polychlorinated biphenyls (PCBs) are a typical persistent organic pollutant with an aromatic biphenyl skeleton containing one to ten chlorine atoms that can theoretically yield 209 different congeners [1]. PCBS are chemically and thermally stable and have accumulated in soil, sediments, and the atmosphere where they can harm human health and the environment because of their toxicity.

From when they were first produced in the 1930s to when they were banned in the 1990s, about 1.3 million tons of PCBs were produced, and tens of thousands of tons are known to have been released into the environment causing widespread pollution [2]. PCBs are of great concern because of their persistence, high lipophilicity, and adverse effects on organisms. Consequently, numerous studies have investigated the levels of PCBs in various environmental samples, such as sediments [3, 4], indoor air [5], residential carpet dust [6], chicken egg yolks [7], fish and meat [8], and human breast milk [9, 10], blood [11], and adipose tissue [12]. Because PCBs tend to accumulate in the food chain, they present a high risk to human and animal health [13], and complete degradation of PCBs in the environment is essential to avoid this.

Biodegradation of PCBs by microbes has been studied extensively. This process mainly occurs via oxidation by enzymatic catalysis. Since 1973, many aerobic bacteria capable of degrading PCB congeners have been isolated, including *Burkholderia* LB400 [14], *Rhodococcus* sp. strain RHA1 [15], *Alcaligenes eutrophus* H850 [16], and *Enterobacter* sp. LY402 [17]. Biphenyl dioxygenase (BphA) plays a major role in the degradation of PCBs [18]. BphA uses O_2_ and electrons to catalyze the dihydroxylation of an aromatic ring as the initial PCB degradation step and determine the substrate specificity for the overall degradation pathway. Cao et al. [19] analyzed the PCB degradation abilities of BphA1 from *Enterobacter* sp. LY402 experimentally and with molecular simulation. They found that the binding free energies of the PCBs were well matched with the degradation rate constants (*k*) for PCBs with different numbers of chlorine substituents. In other words, the binding free energies of the PCBs decrease as *k* increases. Wu et al. [20] compared the binding free energies of pollutants and receptors, and found that a lower binding free energy indicated higher affinity. Therefore, the binding free energy can be used to evaluate the degradation abilities and analyze the biodegradation of PCBs. Liu et al. [21] studied the relationship between the molecular characteristics and degradation rates of substrates degraded by *Enterobacter* sp. LY402. They determined that the dipole moment of a substrate was positively correlated with its degradation rate, and the stretch-bend energy was negatively correlated with the degradation rates. However, the effectiveness of microbial degradation of highly chlorinated PCBs and specific substituted PCBs is limited [22]. To gain insight into the molecular basis of degradation, the key enzymes involved in the PCB biodegradation process, specifically BphA, have been studied intensively [23–25].

Three-dimensional quantitative structure–activity relationship (3D-QSAR) studies can be used to evaluate the structure–activity relationships of a set of molecules using comparative molecular field analysis (CoMFA) and comparative molecular similarity indices analysis (CoMSIA). These two methods can avoid the insufficiency of the traditional two-dimensional model that is used to characterize the relationships between properties and structures, and have clear physical significance and provide rich information about the molecular field energy [26].

The objective of this study was to reveal the relationships between the binding free energies of BphA with PCBs and the PCBs’ migration, persistence, toxicity, and bioaccumulation. First, molecular docking was used to explore the interactions between BphA and 209 PCB congeners. Next, Pearson correlation analysis was performed to study the relationships between the binding free energies and the molecular weights, migration abilities (octanol–air partition coefficients, *K*_OA_), bioaccumulation (bioconcentration factors, BCF), environmental persistence (half-life, *t*_1/2_), and toxicities (half maximal inhibitory concentration, IC_50_) of the PCBs. Finally, QSAR CoMFA and CoMSIA were used to investigate the relationships between the binding free energies and the structures of the PCBs. These results will provide a theoretical foundation for further elucidation of the degradation and molecular modification of PCBs.

## Materials and methods

### Protein structures

Crystal structures of nine types of BphA (PDB IDs: 1ULJ [27], 1WQL [28], 2YFJ [29], 2YFL [30], 2GBX [31], 2XSH [32], 2E4P [33], 3GZX [34], and 3GZY [34]) used in Surflex-Dock were obtained from the Protein Data Bank (http://www.rcsb.org/pdb/). The catalytic activities these BphA were different. The active sites were determined by the amino acid residues in the region where catalytic activity occurred. As different types of BphA may contain multiple active sites, all active sites were docked into the 209 PCBs to ensure the molecular docking was accurate.

### Molecular docking

The molecular docking was performed using the Sybyl-x 2.0 molecular modeling package (Tripos Inc., St. Louis, MO) running on a Windows 7 32-bit workstation. The Surflex-Dock method in the Sybyl package was used to carry out molecular docking simulations to dock the ligands into a receptor’s ligand binding site and represent the interaction strength. 209 PCBs were minimized under the Tripos force field with MMFF94 [35] atomic partial charges by the Powell method, with a maximum iteration of 10,000 to reach a convergence gradient value of 0.001 kcal mol^−1^ Å. Before docking, the natural ligand and structural water molecules were removed from the crystal structure. Using the Biopolymer module, polar hydrogen atoms were added into the standard geometry. Kollman all-atom charges were assigned to protein atoms [36]. In this process, ligands were automatically docked into the binding site of the protein using a ProtoMol-based approach [37] with a patented search engine and an empirical scoring function. We applied the automatic docking method. Based on the software protocol of Surflex-Dock, ProtoMol was produced. “Proto Bloat” and “Proto Thresh” are two important factors that greatly affect the size and extent of the ProtoMol. “Proto Bloat” determines how far ProtoMol extends outside of the concavity, while “Proto Thresh” represents how far ProtoMol extends into the concavity of the target site [38]. In this process, for all ProtoMols produced, “Proto Thresh” was set to 0.5 and “Proto Bloat” was set to 1. All other parameters were at the default settings. Then, a binding pocket was generated. Successful molecular docking is indicated by a root mean square deviation of less than 2 Å [39].

According to the total docking scores, the first twenty conformations of every ligand were saved. The best conformations of the ligands were analyzed for their binding interactions [40]. In the molecular docking process, the receptor protein was considered to be rigid, and the ligand compounds were regarded as being flexible [36].

Surflex-Dock’s scoring function, which contains hydrophobic, polar, repulsive, entropic, and solvation terms, was trained to estimate the dissociation constant (*K*_d_) expressed as − log(*K*_d_) unit [41]. The docking results were ranked in a molecular spreadsheet. The conformer with the highest score was taken as the docking result [20]. For a better comparison between the binding free energies of the PCBs and BphA, the binding scores were converted to binding free energies (kcal/mol). The binding free energy was calculated as follows: where RT = 0.59 kcal/mol [38]:

1 $${\text{Free}}\; {\text{energy}}\; {\text{of}}\; {\text{binding}} = {\text{RT}} \times \ln^{{10^{{ - {\text{pKd}}}} }}$$

RT = 0.59 kcal/mol.

### 3D-QSAR analysis

The calculated binding free energies for 209 PCBs and BphA were used for modeling. CoMFA and CoMSIA methods were applied to build 3D-QSAR models using the structural parameters as independent variables and the binding free energy as the dependent variable to obtain the relationship between the binding free energy and molecular structure. In construction of the 3D-QSAR model, molecular alignment is an important step [42]. In this approach, decachlorobiphenyl, which had the largest binding free energy, was used as the template to align the remaining PCBs. The 209 PCBs were well aligned.

#### CoMFA and CoMSIA analysis

The CoMFA model includes steric and electrostatic descriptor fields. A 3D cubic lattice with a grid spacing of 2 Å and a sp^3^ carbon probe atom was created to calculate the CoMFA descriptor fields. The distance dependent dielectric constant was set at 1.0. The default energy cutoff value was set at 30 kcal mol^−1^. The CoMSIA model was based on the CoMFA model, which was composed of hydrophobic, hydrogen bond donor, and hydrogen bond acceptor fields. A sp^3^ carbon carrying a charge of + 1.0 was used as the probe atom to generate the CoMSIA descriptor fields. The default value of 0.3 was used as the attenuation factor α [43]. CoMSIA uses a Gaussian-type distance dependence to measure the relative attenuation of the field position of each atom in the lattice, and leads to much smoother sampling of the fields around the molecules than CoMFA. Compared with the CoMFA model, CoMSIA can avoid inherent defects, but the results are not necessarily good [44]. Hence, both methods (CoMFA and CoMSIA) can be used to verify and complement each other to obtain a reliable prediction model.

#### Partial least squares regression

Partial least squares regression analysis was applied to establish linear correlations between the binding free energies and the CoMFA and CoMSIA descriptors. Cross-validation with the leave-one-out method was carried out to yield the square of the cross-validation coefficient (*q*^2^) and the optimum number of components (*N*). Then, non-cross-validation analysis was performed with *N* and a column filter of 2.0 kcal mol^−1^ to produce regression models for CoMFA and CoMSIA and accelerate the analysis and decrease the noise. The 3D colored contour maps represent the relationship between the binding free energies and each molecular field.

### Pearson correlation analysis

Pearson correlation analysis was performed using SPSS software to study the relationship between the binding free energies and the molecular weights, *K*_OA_, BCF, *t*_1/2_, and IC_50_ values of the PCBs. The *K*_OA_ [45], BCF [46], *t*_1/2_ [47], and IC_50_ [48] values were obtained directly from the literature. The binding free energies were significantly correlated to the molecular weight, *K*_OA_, BCF, *t*_1/2_, and IC_50_ values when the *P* value was less than 0.01. The binding free energy was significantly correlated to the molecular weight, *K*_OA_, BCF, *t*_1/2_, and IC_50_ when *P* was more than 0.01 but less than 0.05. The binding free energy was not significantly correlated to the molecular weight, *K*_OA_, BCF, *t*_1/2_, or IC_50_ when *P* was more than 0.05.

## Results and discussion

### Comparative analysis of affinities between PCBs and BphA

**Fig. 1:**
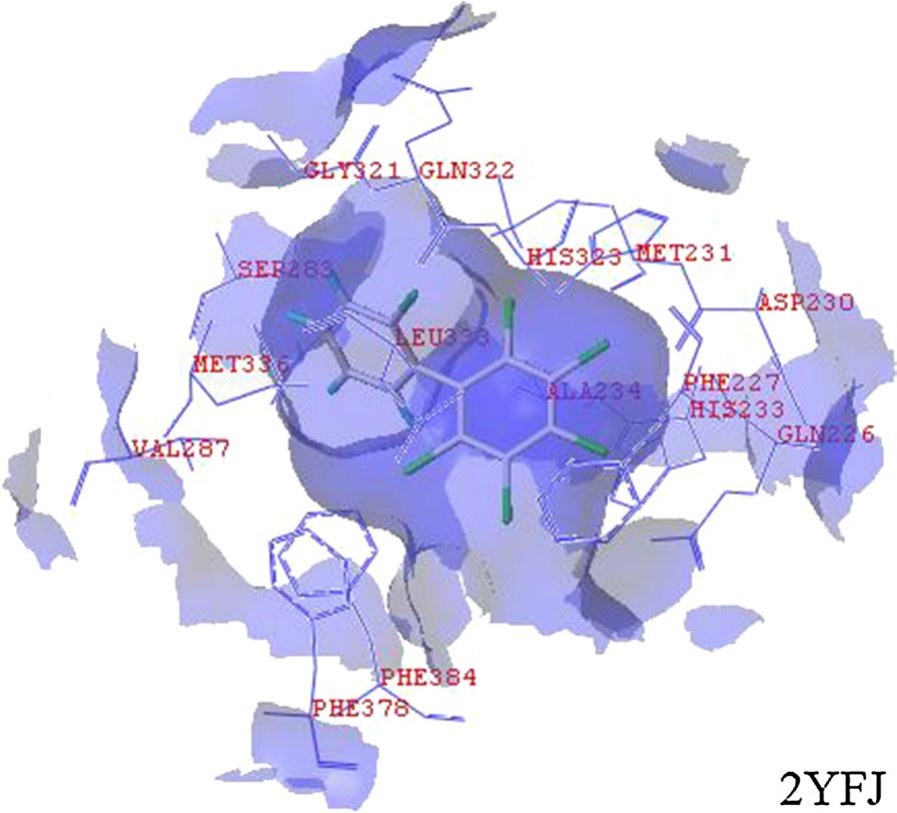
The docking results for 2,2′,3,4,5,6-hexachlorobiphenyl and 2YFJ as examples

**Fig. 2:**
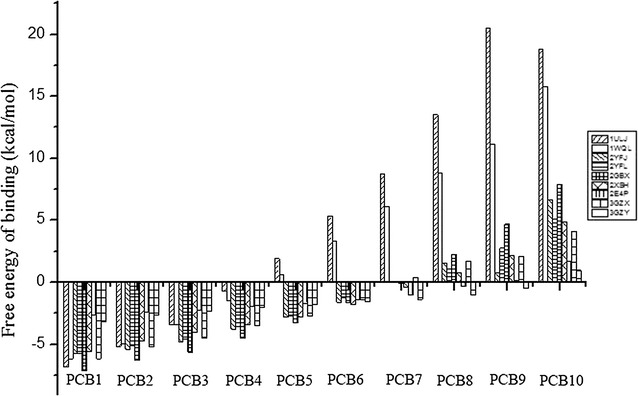
The binding free energies of PCBs classified according to their number of Cl atoms

The root mean square deviation range was 0.0154–0.9753, with all values less than 2 Å, indicating that Surflex-Dock was reliable in causing reappearance of the binding pattern of the ligand, and the parameter setting for molecular docking was appropriate. The docking results are shown in Fig. 1. The binding free energy was calculated using Eq. (1) and was used to evaluate the affinity between the ligand and receptor. Low binding free energies indicate high affinity or catalytic activity [20]. The degradation rate constants of the PCBs decreased as binding free energy increased (i.e., the binding affinity between the ligand and receptor decreased) [19]. As different types of BphA may contain multiple active sites, we selected a group from every BphA that had the highest comprehensive scoring function (Fig. 2).

The binding free energies of the PCBs were well matched with the degradation rate constants (*k*) for the different numbers of chlorine substituents. A lower binding free energy represents stronger binding affinity or catalytic activity [20]. Both 1ULJ and 1WQL could degrade the isomers from chlorobiphenyl to tetrachlorobiphenyl (Fig. 2). The PCB degradation rates of 1ULJ were higher than those of 1WQL for all isomers except tetrachlorobiphenyl, which had a higher degradation rate with 1WQL than 1ULJ. Both 3GZX and 2YFL could degrade the isomers from chlorobiphenyl to hexachlorobiphenyl. The PCB degradation rate of 3GZX was higher than that of 2YFL. 2GBX, 2XSH, and 2YFJ degraded the isomers from chlorobiphenyl to pentachlorobiphenyl with PCB degradation rates decreasing in the following order: 2GBX > 2YFJ > 2XSH. For hexachlorobiphenyl and heptachlorobiphenyl, the degradation rates with 2XSH were higher than those with 2GBX and 2YFJ. 2E4P was able to degrade the isomers from chlorobiphenyl to octachlorobiphenyl, and 3GZY degraded the isomers from chlorobiphenyl to nonachlorobiphenyl.

Although all of the types of BphA could degrade the less-chlorinated PCBs (one to four Cl atoms), the degradation rates were different. The PCB degradation rates of the different types of BphA increased in the following order: 2E4P < 3GZY < 1WQL < 1ULJ < 2XSH < 2YFL < 2YFJ < 3GZX < 2GBX. 3GZY was able to degrade highly chlorinated PCBs (five to nine Cl atoms), but the degradation rates were not high. For pentachlorobiphenyl and hexachlorobiphenyl, the degradation rates of seven types of BphA were in the following order: 2GBX > 2XSH > 2YFJ > 2YFL > 3GZX > 3GZY > 2E4P. For heptachlorobiphenyl, the degradation rates of five types of BphA increased in the following order: 2YFJ < 2GBX < 2XSH < 2E4P < 3GZY. The degradation rate for octachlorobiphenyl was higher with 3GZY than with 2E4P. Our results correspond with those from previous studies that found that low binding free energies were an indicator of high affinity [20].

Chen et al. [45] studied the application of PCB industrial products in practical production. Their results showed that isomers from trichlorobiphenyl to heptachlorobiphenyl accounted for the majority of the commercial mixtures of PCBs, and chlorobiphenyl and dichlorobiphenyl also contributed a substantial proportion [45]. In the environment, the isomers from trichlorobiphenyl to heptachlorobiphenyl are the primary PCBs that undergo degradation. In this study, 2GBX, 2XSH, 2YFJ, 2E4P, and 3GZY had the ability to degrade the isomers from trichlorodiphenyl to heptachlorobiphenyl, and the PCB degradation rates were in the following order: 2GBX > 2YFJ > 2XSH > 3GZY > 2E4P. For single PCBs, 2GBX had the highest degradation rates for the isomers from chlorobiphenyl to pentachlorobiphenyl, 2XSH had the best ability to degrade hexachlorobiphenyl, and 3GZY had the highest degradation rates for isomers from heptachlorobiphenyl to nonachlorobiphenyl. In the microbial degradation system for PCBs, the biodegradation ratios of the PCBs were maximized with addition of 2GBX, 2XSH, and 3GZY.

### Correlation analysis of the relationships between the binding free energies and the persistent organic pollutant characteristics of the PCBs

In this study, Pearson correlation analysis was used to study the relationships between the binding free energies and the molecular weight, *K*_OA_, BCF, *t*_1/2_, and IC_50_ values for the PCBs (Table 1).

**Table 1:** Pearson correlation analysis between the binding free energies and the properties of the PCBs

	BphA	1ULJ	1WQL	2YFJ	2YFL	2GBX	2XSH	2E4P	3GZX	3GZY
Molecular weight	r	0.982**	0.989**	0.945**	0.983**	0.974**	0.975**	0.946**	0.992**	0.950**
	Sig.	0	0	0	0	0	0	0	0	0
	N	10	10	10	10	10	10	10	10	10
*K*_OA_	r	0.882**	0.744**	0.712**	0.756**	0.864**	0.734**	0.421**	0.744**	0.514**
	Sig.	0	0	0	0	0	0	0	0	0
	N	209	209	209	209	209	209	209	209	209
BCF	r	0.774**	0.682**	0.640**	0.707**	0.743**	0.660**	0.593**	0.592**	0.459**
	Sig.	0	0	0	0	0	0	0	0	0
	N	209	209	209	209	209	209	209	209	209
*t*_1/2_	r	0.591**	0.609**	0.565**	0.563**	0.549**	0.527**	0.540**	0.572**	0.404**
	Sig.	0	0	0	0	0	0	0	0	0
	N	209	209	209	209	209	209	209	209	209
IC_50_	r	− 0.117	0.090	− 0.080	− 0.113	− 0.163	0.226	0.303	0.060	0.062
	Sig.	0.593	0.684	0.717	0.606	0.458	0.300	0.160	0.786	0.780
	N	23	23	23	23	23	23	23	23	23

“r” represents “Pearson correlation coefficient, the bigger the r, the stronger the correlation”; “sig.” means “significant, if sig < 0.05, indicating the significant correlation”; “N” represents “sample size”;“**” means “the coefficient statistically significant was at P = 0.01” (n = 209)

The Pearson correlation analysis revealed that the binding free energies were significantly correlated to the molecular weight, *K*_OA_, BCF, and *t*_1/2_ values (*P* = 0 < 0.01), but not significantly correlated to the IC_50_ values (*P* < 0.05).

The binding free energies between the PCBs and BphA gradually increased as the number of Cl atoms increased (Fig. 2), that is, the binding free energy gradually increased with increasing molecular weight. This indicted a positive correlation between the binding free energy and molecular weight. Large *K*_OA_ values for the PCBs imply they have stronger migration abilities [45]. Strong migration abilities may suggest that PCBs are not easily degraded in the environment because the atmospheric conditions are not suitable for biodegradation. Because the binding free energy was negatively correlated with the degradation ability, low degradation ability implied the binding free energy was strong. Therefore, the binding free energy was positively correlated with the *K*_OA_ values of the PCBs. Large BCF values implied that the PCBs strongly bioaccumulated in the body [44]. With gradual accumulation in the food chain, the levels of PCBs in the body will gradually increase, and this will be accompanied by a decrease in the amount of PCBs in the environment. This is not beneficial for PCB degradation in the environment by microorganisms. Hence, PCBs are more difficult to degrade following an increase in the binding free energy, which shows that the binding free energy is positively correlated with the BCF. Large *t*_1/2_ values for the PCBs imply that they are persistent in the environment [45]. Hence, PCBs are more difficult to degrade following an increase in the binding free energy, which agrees with earlier studies.

### CoMFA and CoMSIA contour analysis

The *q*^2^ from the CoMFA models had a range of 0.585–0.908 (> 0.5), the *r*^2^ range was 0.717–0.948 (> 0.6) [49], the SEE range was 0.357–1.380, and the *F* range was 32.668–269.003 (Table 2). Therefore, the CoMFA model was robust. For the CoMSIA models, the *q*^2^ range was 0.521–0.897 (> 0.5), the *r*^2^ range was 0.630–0.930 (> 0.6) [49], the SEE range was 0.394–1.750, and the *F* range was 20.393–197.637. Therefore, the CoMFA model was robust. The contribution rates of the electrostatic fields to the CoMFA and CoMSIA models were in the range of 0.609–0.848, which showed that the electrostatic field was important in each of the two models. Chen et al. [45] found that the electrostatic field played an important role in determining the log*K*_OA_ values of PCBs, Liu et al. [44] determined that the electrostatic descriptor was a primary factor governing the logBCF, and Xu et al. [45] found that electrostatic interaction was crucial in determining the *t*_1/2_ values. These results are consistent with the positive correlations between the binding free energies and the *K*_OA_, BCF, and *t*_1/2_ values for the PCBs, which can be indirectly explained by the correlation between the binding free energies and the K_OA_, BCF, and *t*_1/2_ values for the PCBs from the molecular field perspective. The binding free energies between the nine types of BphA and 209 PCBs are shown in Table S1 (shown in the Additional file 1 available to the readers).

**Table 2:** Statistical parameters of the CoMFA and CoMSIA models

Model	1ULJ	1WQL	2YFJ	2YFL	2GBX	2XSH	2E4P	3GZX	3GZY
CoMFA									
n	18	10	14	15	20	10	7	10	15
q^2^	0.908	0.902	0.818	0.826	0.896	0.82	0.691	0.873	0.585
r^2^	0.948	0.931	0.881	0.892	0.948	0.862	0.770	0.905	0.717
SEE	1.380	1.195	0.804	0.728	0.681	0.700	0.357	0.742	0.409
F	192.403	269.003	102.590	106.547	169.704	123.646	95.879	189.390	32.668
S	0.381	0.391	0.341	0.302	0.336	0.391	0.387	0.389	0.319
E	0.619	0.609	0.659	0.698	0.664	0.609	0.613	0.611	0.681
CoMSIA									
n	15	18	10	20	8	20	3	19	16
q^2^	0.897	0.897	0.799	0.801	0.869	0.811	0.681	0.855	0.521
r^2^	0.915	0.930	0.825	0.865	0.888	0.856	0.714	0.905	0.630
SEE	1.750	1.236	0.964	0.828	0.965	0.733	0.394	0.763	0.469
F	138.685	139.531	93.571	59.999	197.637	55.866	170.659	94.295	20.393
S	0.024	0.030	0.009	0.027	0.009	0.025	0.008	0.027	0.031
E	0.769	0.681	0.837	0.716	0.831	0.721	0.848	0.709	0.677
H	0.207	0.289	0.154	0.257	0.160	0.254	0.144	0.264	0.292
D	–	–	–	–	–	–	–	–	–
A	–	–	–	–	–	–	–	–	–

*N*, optimum number of components; *r*^2^, correlation coefficient; *q*^2^, cross-validated val; SEE, standard error of the estimate; *F*, Fischer’s test value; S, steric; E, electrostatic; H, hydrophobic; D, hydrogen bond donor; A, hydrogen bond acceptor

**Fig. 3:**
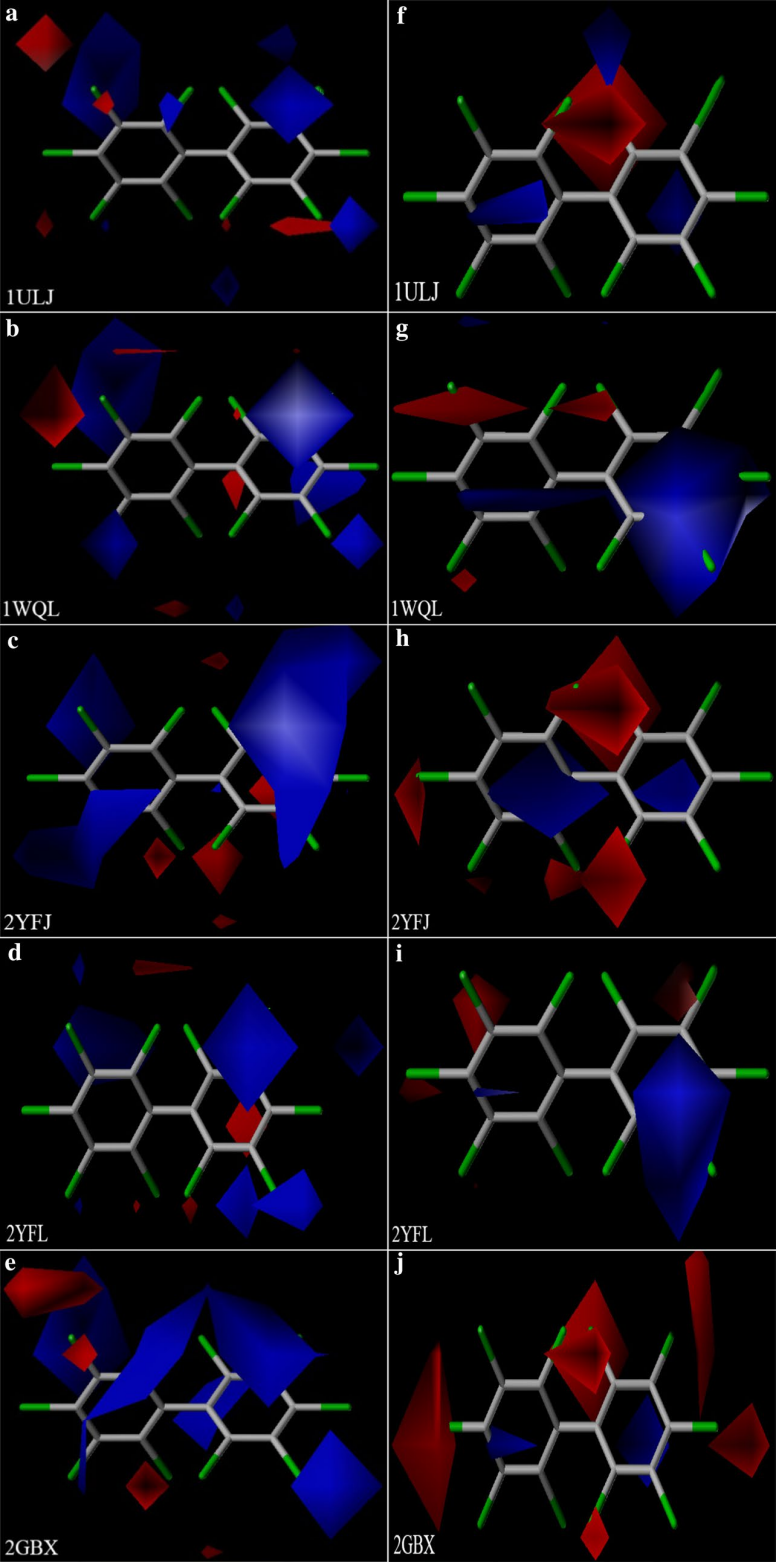
Electrostatic contour maps of CoMFA (**a**–**e**) and CoMSIA (**f**–**j**) models

**Fig. 4:**
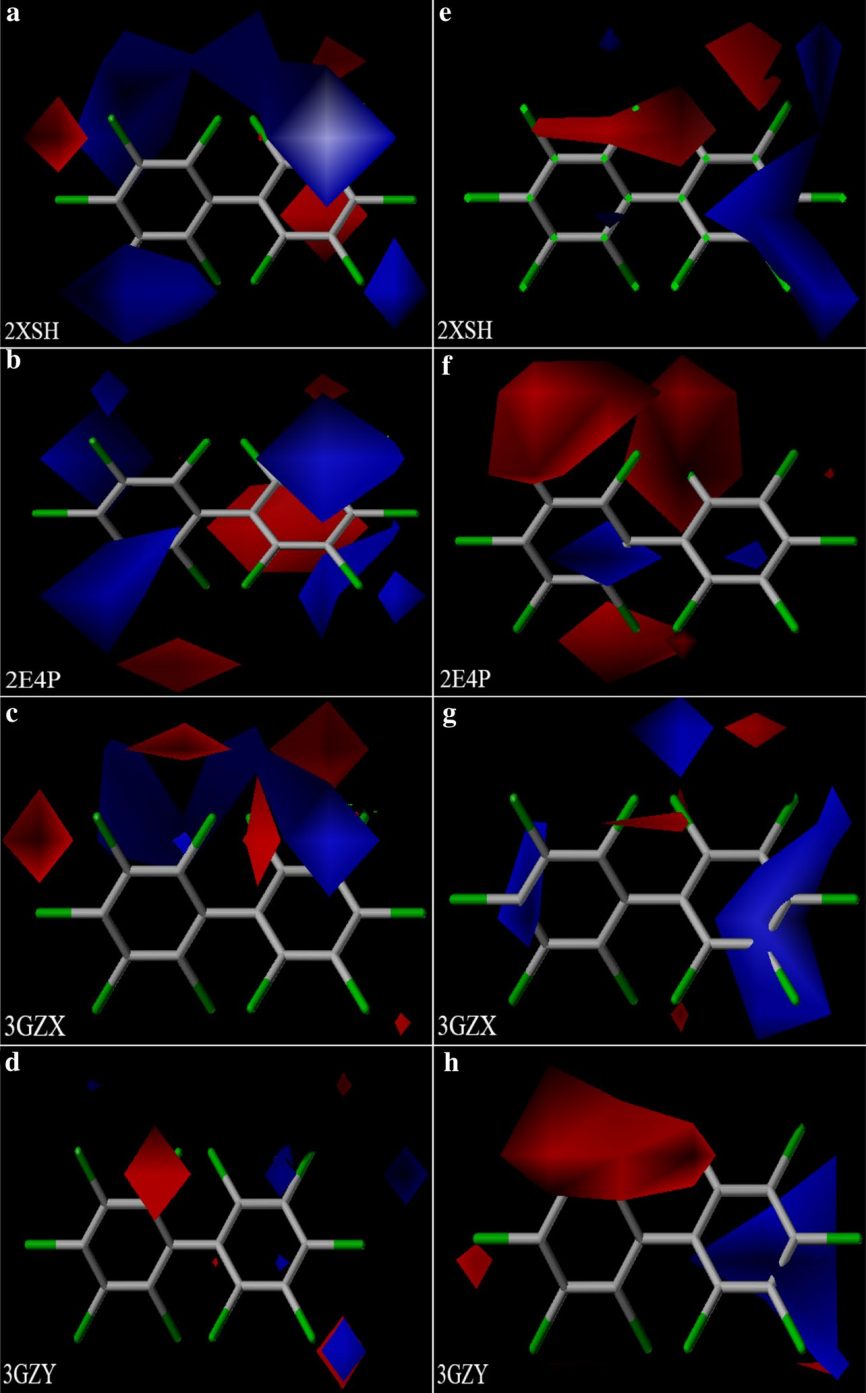
Electrostatic contour maps of CoMFA (**a**–**d**) and CoMSIA (**e**–**h**) models

The most interesting feature of CoMFA and CoMSIA models is the visualization of the results as 3D contour plots. We can observe the contour of the 3D space around the molecule, in which changes in the physicochemical properties are predicted to increase or decrease the effectiveness of the contour [50]. The results for the CoMFA and CoMSIA models are illustrated in field contribution graphs using the standard deviation times coefficient field. The contour maps of the two models (Figs. 3, 4) show the electrostatic fields with decachlorobiphenyl as a template. These contour maps showed 80 and 20% contributions for favorable and unfavorable regions, respectively. The electrostatic field is represented by blue and red contours, with blue regions indicating the electropositive groups close to these regions are favorable to the binding free energy, and the red regions indicate the electronegative groups near these regions can enhance the binding free energy. By contrast, the binding free energy decreased when electropositive groups were introduced in the red region or electronegative groups in the blue region.

Although the electrostatic field was identified as a major factor affecting the binding free energies between BphA and the PCBs, there were some differences in the electrostatic contour maps (Figs. 3, 4, Table 3).

**Table 3:** Results for the electrostatic contour maps of the CoMFA and CoMSIA models

Position	Electrostatic fields	2	3	4	5	6	2′	3′	4′	5′	6′
1ULJ	Electropositive	+			+			+			
	Electronegative	−	−				−			−	−
1WQL	Electropositive				+			+	+	+	
	Electronegative	−	−		−		−				
2YFJ	Electropositive		+		+			+		+	
	Electronegative	−	−	−	−	−					−
2YFL	Electropositive				+			+		+	
	Electronegative	−	−	−		−		−			−
2GBX	Electropositive	+			+			+			
	Electronegative	−	−	−		−	−	−	−		−
2XSH	Electropositive				+			+		+	
	Electronegative	−	−				−				
2E4P	Electropositive		+		+			+		+	
	Electronegative	−	−			−	−				−
3GZX	Electropositive	+						+	+	+	
	Electronegative	−	−				−			−	−
3GZY	Electropositive							+	+	+	
	Electronegative	−	−	−			−				

“+” represents electropositive groups introduced in this position, “−” represents electronegative groups introduced in this position

The binding free energies between the PCBs and the nine types of BphA increased simultaneously with substituents possessing electropositive groups at the 3′-position of the B ring or electronegative groups at the 2- and 3-positions of the A ring. In the electrostatic fields of 1WQL, 2YFJ, 2YFL, 2XSH, and 2E4P, electropositive groups were desirable at the 3′- and 5-positions to increase the binding free energies. In the electrostatic fields of 2GBX and 3GZX, the electropositive groups close to the regions of the 2- and 3′-positions increased the binding free energies between the PCBs and BphA. Introducing electropositive groups at the 2-, 3′-, and 5-positions increased the binding free energies between the PCBs and 1ULJ. The binding free energies between the PCBs and 3GZY increased with substituents possessing electropositive groups at the 3′-position of the B ring.

In the electrostatic fields of 1ULJ and 3GZX, introducing electronegative groups to the 2-, 3-, and 6-positions of the A ring increased the binding free energies. The binding free energies between PCB-24 and PCB-5 could be deciphered using this contour. In the electrostatic fields of 2YFJ and 2GBX, electronegative groups near the 2-, 3-, 3′-, 4-, 6-, and 6′-positions were favorable for enhancing the binding free energies, as shown by the higher binding free energy for PCB-109 than for PCB-55 or PCB-59. The binding free energies between the PCBs and 1WQL were enhanced with electronegative groups at the 2-, 3-, and 5-positions of the A ring. For example, the binding free energy of PCB-23 was higher than those of PCB-6 and PCB-9. Electronegative groups were desirable at the 2-, 3-, and 4-positions of the A ring and increased the binding free energies between the PCBs and 3GZY, as shown by the higher binding free energy for PCB-21 than for PCB-7 or PCB-12. Introducing electronegative groups to the 2- and 3-positions of the A ring increased the binding free energies between the PCBs and 2XSH. The same applied for the binding free energies between PCB-5 and PCB-2. The binding free energies between the PCBs and 2E4P increased on introduction of substituents with electronegative groups at the 2-, 3-, 6-, and 6′-positions. An example of this was the high binding free energy of PCB-5 compared with those of PCB-1 and PCB-2. Electronegative groups close to the regions of the 2-, 4-, 5-, 6-, and 6′-positions increased the binding free energies between the PCBs and 2YFJ, as shown by the higher binding free energy of PCB-30 than for PCB-7 or PCB-10.

The log*K*_OA_ increased on introduction of substituents with electropositive groups at the 3′- and 6-positions or electronegative groups at the 2-, 3-, 3′- and 5-positions [45]. The log*K*_OA_ and the binding free energies between the PCBs and the nine types of BphA increased simultaneously on introduction of substituents with electropositive groups at the 3′-position of the B ring or electronegative groups at the 2-position of the A ring. In the electrostatic fields of 1ULJ, 2XSH, 2E4P, 3GZX, 3GZY, 1WQL, 2YFL, 2GBX, and *K*_OA_, introducing electronegative groups at the 3-position of the A ring increased the binding free energies and the *K*_OA_ simultaneously. In the electrostatic fields of 2YFJ, 1WQL, and *K*_OA_, electronegative groups near the 5-position were favorable for enhancing the binding free energies and the *K*_OA_ simultaneously. Therefore, using molecular modeling, we proved that the binding free energies were positively correlated with the migration abilities of the PCBs.

The BCF values increased on introduction of substituents with electropositive groups at the 2-, 3′-, 5-, and 6-positions or electronegative groups at the 3-, 4-, and 5-positions of the A ring [44]. The BCF values of the PCBs and the binding free energies between the PCBs and BphA increased simultaneously on introduction of substituents with electropositive groups at the 3′-position of the B ring or electronegative groups at the 3-position of the A ring. In the electrostatic fields of 1ULJ, 3GZX, 3GZY, 2GBX, and BCF, electropositive groups were desirable at the 3′-positions of the B ring for increases in the binding free energies and the BCF values. In the electrostatic fields of 1WQL, 2YFJ, 2YFL, 2XSH, 2E4P and BCF, introducing electropositive groups to the 3′- and 5-positions increased the binding free energies and BCF values simultaneously. In the electrostatic fields of 1ULJ, 3GZX, 2XSH, 2E4P, and BCF, electronegative groups close to the regions of the 3-position of the A ring increased the binding free energies and the BCF values. In the electrostatic fields of 2YFJ, 2GBX, 3GZY, and BCF, electronegative groups near the 3- and 4-positions of the A ring were favorable for enhancing the binding free energies and the BCF values simultaneously. The introduction of electronegative groups to the 3- and 5-positions of the A ring increased the BCF values and the binding free energies between PCBs and 1WQL. Electronegative groups were desirable at the 4- and 5-positions of the A ring to increase the BCF values of the PCBs and the binding free energies between the PCBs and 2YFJ. Therefore, the binding free energies were positively correlated with the bioaccumulation of PCBs, which was proven by molecular modeling.

It was determined that electronegative groups close to the regions of the 3-, 3′-, 5-, 6-, and 6′-positions increased the *t*_1/2_ values of the PCBs [45]. The *t*_1/2_ values of the PCBs and the binding free energies between the PCBs and the nine types of BphA increased simultaneously on introduction of substituents with electronegative groups at the 3-position of the A ring. In the electrostatic fields of 2YFL, 2GBX, and *t*_1/2_, electronegative groups were desirable at 3-, 3′-, 6-, 6′-positions to increase the binding free energies and *t*_1/2_ values. Electronegative groups close to the regions of the 3- and 5-positions of the A ring increased the *t*_1/2_ values and binding free energies between the PCBs and 1WQL. Electronegative groups near the 3-, 5-, 6-, and 6′-positions were favorable for simultaneously enhancing the *t*_1/2_ values and the binding free energies between PCBs and 2YFJ. Introducing electronegative groups at the 3- and 6′-positions increased the t_1/2_ values and the binding free energies between the PCBs and 2E4P. Electronegative groups were desirable at the 3-, 6-, and 6′-positions to increase the *t*_1/2_ values of the PCBs and the binding free energies between the PCBs and 3GZX. Molecular modeling showed that the binding free energies were positively correlated with the persistence of the PCBs.

## Conclusions

The main research conclusions are as follows:

1. Among the nine types of BphA, 2GBX gives the highest PCB degradation rate.
2. The binding free energy is highly significantly correlated with the molecular weight, *K*_OA_, BCF, and *t*_1/2_ values, but is not significantly correlated with the IC_50_ values.
3. The electrostatic descriptors play a more significant role than steric descriptors and hydrophobic descriptors. This is consistent with literature findings of the electrostatic field as a major factor governing the *K*_OA_, *t*_1/2_, and BCF values. Therefore, that the influence of molecular structure on the binding free energies, *K*_OA_, *t*_1/2_, and BCF values is consistent.
4. In an electrostatic field, introducing electropositive groups in the 3-position or electronegative groups in the 3′-position of a PCB significantly reduces the binding free energies, molecular weights, *K*_OA_, BCF, and *t*_1/2_ values. Therefore, the binding free energies are correlated with the molecular weights, *K*_OA_, BCF, and *t*_1/2_ values of the PCBs because of structural features of the molecules.

## Additional file

**Additional file 1: Table S1.** The binding free energies between nine types of BphA and 209 PCBs.
