# Supplementary material for: Relationship between the binding free energy and PCBs’ migration, persistence, toxicity and bioaccumulation using a combination of the molecular docking method and 3D-QSAR
Source: Chem Cent J. 2018 Feb 23;12:20. doi: 10.1186/s13065-018-0389-2 (PMC5825354; doi:10.1186/s13065-018-0389-2)
Supplement: Supplementary file 1 — Additional file 1: Table S1. The binding free energies between nine types of BphA and 209 PCBs. [file 13065_2018_389_MOESM1_ESM.docx]

Table S1. The binding free energies between nine types of BphA and 209 PCBs

| No. | 2GBX | 2YFJ | 1ULJ | 1WQL | 3GZX | 3GZY | 2E4P | 2XSH | 2YFL |
| --- | --- | --- | --- | --- | --- | --- | --- | --- | --- |
| 1 | -7.322 | -6.204 | -6.761 | -6.670 | -6.276 | -3.165 | -6.473 | -5.371 | -4.819 |
| 2 | -6.648 | -6.050 | -6.557 | -6.181 | -6.276 | -3.532 | -6.229 | -5.330 | -4.881 |
| 3 | -6.652 | -5.497 | -5.688 | -5.665 | -5.937 | -2.785 | -5.801 | -4.796 | -4.293 |
| 4 | -6.367 | -5.289 | -5.946 | -5.719 | -5.081 | -3.125 | -5.400 | -4.642 | -4.262 |
| 5 | -6.698 | -5.728 | -4.995 | -5.964 | -5.719 | -2.540 | -5.842 | -4.741 | -4.191 |
| 6 | -6.630 | -5.828 | -5.882 | -5.774 | -5.312 | -2.608 | -5.543 | -4.565 | -4.076 |
| 7 | -6.313 | -5.430 | -4.922 | -4.714 | -5.638 | -2.771 | -5.176 | -4.374 | -3.974 |
| 8 | -6.131 | -5.651 | -4.827 | -4.103 | -4.850 | -2.377 | -4.476 | -3.777 | -3.427 |
| 9 | -6.539 | -5.126 | -5.887 | -4.415 | -5.244 | -2.391 | -4.830 | -4.017 | -3.610 |
| 10 | -6.376 | -4.814 | -4.470 | -4.415 | -4.198 | -2.242 | -4.307 | -3.618 | -3.274 |
| 11 | -6.562 | -4.542 | -3.903 | -5.747 | -4.986 | -1.916 | -5.366 | -4.216 | -3.641 |
| 12 | -5.226 | -5.425 | -3.251 | -6.195 | -6.616 | -3.288 | -6.405 | -5.366 | -4.847 |
| 13 | -5.425 | -4.651 | -5.131 | -5.719 | -4.537 | -3.070 | -5.128 | -4.442 | -4.099 |
| 14 | -5.728 | -4.646 | -3.423 | -3.478 | -5.013 | -2.201 | -4.245 | -3.564 | -3.223 |
| 15 | -5.099 | -4.841 | -2.024 | -3.057 | -4.755 | -3.546 | -3.906 | -3.786 | -3.726 |
| 16 | -6.304 | -5.235 | -4.089 | -5.027 | -5.027 | -1.766 | -5.027 | -3.940 | -3.396 |
| 17 | -5.339 | -4.098 | -4.388 | -2.744 | -4.850 | -2.540 | -3.797 | -3.378 | -3.169 |
| 18 | -5.955 | -4.465 | -4.959 | -4.374 | -4.524 | -2.214 | -4.449 | -3.704 | -3.332 |
| 19 | -5.547 | -3.229 | -3.188 | -4.415 | -2.880 | -1.956 | -3.648 | -3.084 | -2.802 |
| 20 | -6.684 | -4.447 | -3.559 | -4.578 | -4.850 | -2.921 | -4.714 | -4.116 | -3.817 |
| 21 | -5.543 | -5.393 | -2.196 | -4.062 | -6.358 | -2.744 | -5.210 | -4.388 | -3.977 |
| 22 | -5.298 | -3.645 | -2.853 | -3.546 | -4.510 | -2.269 | -4.028 | -3.442 | -3.148 |
| 23 | -5.724 | -4.605 | -1.897 | -3.763 | -4.483 | -1.589 | -4.123 | -3.279 | -2.856 |
| 24 | -6.530 | -4.542 | -3.704 | -2.201 | -4.388 | -2.160 | -3.294 | -2.916 | -2.727 |
| 25 | -5.135 | -3.623 | -4.144 | -3.804 | -4.796 | -2.296 | -4.300 | -3.632 | -3.298 |
| 26 | -6.385 | -3.786 | -3.935 | -3.383 | -4.755 | -2.038 | -4.069 | -3.392 | -3.053 |
| 27 | -6.159 | -3.944 | -2.473 | -1.630 | -2.975 | -2.242 | -2.303 | -2.282 | -2.272 |
| 28 | -5.158 | -4.642 | -1.168 | -1.739 | -4.673 | -3.885 | -3.206 | -3.433 | -3.546 |
| 29 | -4.687 | -4.370 | -1.105 | -3.980 | -4.021 | -2.377 | -4.001 | -3.460 | -3.189 |
| 30 | -4.796 | -3.754 | -2.907 | -2.540 | -3.369 | -2.500 | -2.955 | -2.803 | -2.727 |
| 31 | -5.040 | -4.470 | -2.808 | -2.989 | -4.578 | -2.907 | -3.783 | -3.491 | -3.345 |
| 32 | -5.570 | -4.297 | -0.897 | -0.408 | -4.198 | -1.535 | -2.303 | -2.047 | -1.919 |
| 33 | -5.262 | -5.276 | -2.613 | -4.728 | -5.149 | -2.744 | -4.938 | -4.207 | -3.841 |
| 34 | -5.008 | -4.225 | -2.531 | -4.252 | -4.334 | -2.038 | -4.293 | -3.541 | -3.165 |
| 35 | -4.479 | -4.320 | -1.762 | -5.814 | -4.361 | -2.554 | -5.088 | -4.243 | -3.821 |
| 36 | -4.941 | -3.188 | -0.842 | -2.785 | -4.578 | -1.562 | -3.682 | -2.975 | -2.622 |
| 37 | -4.474 | -4.438 | -1.458 | -4.578 | -4.836 | -3.763 | -4.707 | -4.393 | -4.235 |
| 38 | -4.768 | -4.705 | -2.350 | -1.712 | -5.135 | -2.146 | -3.423 | -2.998 | -2.785 |
| 39 | -4.728 | -4.551 | -1.662 | -2.282 | -4.116 | -1.630 | -3.199 | -2.676 | -2.415 |
| 40 | -6.041 | -3.795 | -2.703 | -2.513 | -3.899 | -2.364 | -3.206 | -2.925 | -2.785 |
| 41 | -5.706 | -4.660 | -1.309 | -3.070 | -5.312 | -2.214 | -4.191 | -3.532 | -3.203 |
| 42 | -4.633 | -2.513 | -2.128 | -3.030 | -3.342 | -2.038 | -3.186 | -2.803 | -2.612 |
| 43 | -5.022 | -3.446 | -1.512 | -4.062 | -3.546 | -1.589 | -3.804 | -3.066 | -2.697 |
| 44 | -6.009 | -3.944 | -3.197 | -2.540 | -3.654 | -2.092 | -3.097 | -2.762 | -2.595 |
| 45 | -5.629 | -3.804 | -2.323 | -1.236 | -3.165 | -1.820 | -2.201 | -2.074 | -2.011 |
| 46 | -5.285 | -3.333 | -1.128 | -0.883 | -2.079 | -1.481 | -1.481 | -1.481 | -1.481 |
| 47 | -3.926 | -1.540 | 0.439 | -0.706 | -3.315 | -3.451 | -2.011 | -2.491 | -2.731 |
| 48 | -3.931 | -3.179 | -1.005 | -3.532 | -4.361 | -2.051 | -3.947 | -3.315 | -2.999 |
| 49 | -4.130 | -2.577 | -2.350 | -0.788 | -3.817 | -1.970 | -2.303 | -2.192 | -2.136 |
| 50 | -4.076 | -2.242 | -1.349 | -1.494 | -1.956 | -1.888 | -1.725 | -1.780 | -1.807 |
| 51 | -4.510 | -1.721 | -0.552 | 1.141 | -2.962 | -1.657 | -0.910 | -1.159 | -1.284 |
| 52 | -5.538 | -2.744 | -2.617 | 0.068 | -3.695 | -2.133 | -1.814 | -1.920 | -1.973 |
| 53 | -4.777 | -2.328 | -0.466 | 1.712 | -1.304 | -1.685 | 0.204 | -0.426 | -0.740 |
| 54 | -3.220 | -1.603 | 3.106 | 1.576 | 0.231 | -1.698 | 0.903 | 0.036 | -0.397 |
| 55 | -4.601 | -3.342 | -1.250 | -4.144 | -4.537 | -2.513 | -4.340 | -3.731 | -3.427 |
| 56 | -4.365 | -3.618 | 0.177 | -4.035 | -4.171 | -2.513 | -4.103 | -3.573 | -3.308 |
| 57 | -5.036 | -4.483 | 0.149 | -1.888 | -4.551 | -2.323 | -3.220 | -2.921 | -2.771 |
| 58 | -4.388 | -3.514 | 0.965 | -1.046 | -4.565 | -2.133 | -2.805 | -2.581 | -2.469 |
| 59 | -6.118 | -3.623 | -3.414 | -2.649 | -3.600 | -2.065 | -3.125 | -2.771 | -2.595 |
| 60 | -4.714 | -3.016 | 0.489 | -2.038 | -4.565 | -3.777 | -3.301 | -3.460 | -3.539 |
| 61 | -4.180 | -3.867 | 1.780 | -1.087 | -4.714 | -1.875 | -2.900 | -2.559 | -2.388 |
| 62 | -4.460 | -3.822 | 0.163 | -1.861 | -3.600 | -2.405 | -2.731 | -2.622 | -2.568 |
| 63 | -4.990 | -3.568 | 0.901 | -0.217 | -4.470 | -1.644 | -2.343 | -2.110 | -1.994 |
| 64 | -5.253 | -2.984 | 0.312 | -0.584 | -4.239 | -1.372 | -2.411 | -2.065 | -1.892 |
| 65 | -5.054 | -3.614 | 1.576 | -0.014 | -2.717 | -1.739 | -1.365 | -1.490 | -1.552 |
| 66 | -3.926 | -2.948 | 0.009 | -2.309 | -4.918 | -2.799 | -3.614 | -3.342 | -3.206 |
| 67 | -3.822 | -3.437 | -0.448 | -2.894 | -4.089 | -1.250 | -3.491 | -2.744 | -2.371 |
| 68 | -3.396 | -2.776 | -0.856 | -1.657 | -3.587 | -1.956 | -2.622 | -2.400 | -2.289 |
| 69 | -3.990 | -1.929 | -1.449 | -1.073 | -2.568 | -2.106 | -1.820 | -1.916 | -1.963 |
| 70 | -4.094 | -3.564 | -1.096 | -3.043 | -4.279 | -2.092 | -3.661 | -3.138 | -2.877 |
| 71 | -3.808 | -3.111 | 0.725 | 0.190 | -2.771 | -1.997 | -1.291 | -1.526 | -1.644 |
| 72 | -4.882 | -2.839 | -1.010 | -0.639 | -3.138 | -1.929 | -1.888 | -1.902 | -1.909 |
| 73 | -4.198 | -2.237 | 3.034 | 0.367 | -2.309 | -1.793 | -0.971 | -1.245 | -1.382 |
| 74 | -3.251 | -3.858 | 1.644 | -1.196 | -3.464 | -2.255 | -2.330 | -2.305 | -2.293 |
| 75 | -3.138 | -3.156 | 1.997 | -0.326 | -1.875 | -1.386 | -1.100 | -1.196 | -1.243 |
| 76 | -4.651 | -3.827 | 0.367 | -0.666 | -4.714 | -1.943 | -2.690 | -2.441 | -2.316 |
| 77 | -2.436 | -3.387 | 3.487 | -4.442 | -3.831 | -2.622 | -4.137 | -3.632 | -3.379 |
| 78 | -2.676 | -3.197 | 1.875 | -1.807 | -4.076 | -1.698 | -2.941 | -2.527 | -2.320 |
| 79 | -2.405 | -3.396 | 3.256 | -3.125 | -4.252 | -1.698 | -3.688 | -3.025 | -2.693 |
| 80 | -2.201 | -2.998 | 5.208 | -0.027 | -2.350 | -1.196 | -1.189 | -1.191 | -1.192 |
| 81 | -3.541 | -4.642 | 0.005 | -1.481 | -3.165 | -1.997 | -2.323 | -2.214 | -2.160 |
| 82 | -4.162 | -2.699 | 0.571 | -2.880 | -4.497 | -2.106 | -3.688 | -3.161 | -2.897 |
| 83 | -4.642 | -3.134 | 1.657 | -0.543 | -3.858 | -1.793 | -2.201 | -2.065 | -1.997 |
| 84 | -4.891 | -2.785 | -0.851 | 0.149 | -2.405 | -1.141 | -1.128 | -1.132 | -1.134 |
| 85 | -2.916 | -1.205 | 1.703 | -0.136 | -3.410 | -1.834 | -1.773 | -1.793 | -1.803 |
| 86 | -5.620 | -4.207 | -1.635 | -2.636 | -5.325 | -3.451 | -3.980 | -3.804 | -3.716 |
| 87 | -3.138 | -3.306 | 0.072 | -0.666 | -3.967 | -2.160 | -2.316 | -2.264 | -2.238 |
| 88 | -3.428 | -2.586 | 0.806 | -0.584 | -2.826 | -1.807 | -1.705 | -1.739 | -1.756 |
| 89 | -2.762 | -2.359 | 1.340 | 2.065 | -1.848 | -2.119 | 0.109 | -0.634 | -1.005 |
| 90 | -3.482 | -1.055 | 1.399 | -0.815 | -2.975 | -1.617 | -1.895 | -1.802 | -1.756 |
| 91 | -4.171 | -1.997 | 1.490 | 1.440 | -2.146 | -1.997 | -0.353 | -0.901 | -1.175 |
| 92 | -3.514 | -2.219 | -0.136 | 0.706 | -2.717 | -1.522 | -1.005 | -1.177 | -1.263 |
| 93 | -4.451 | -2.767 | 2.146 | 3.016 | -2.011 | -1.426 | 0.503 | -0.140 | -0.462 |
| 94 | -3.039 | -1.494 | 3.496 | 1.168 | -1.725 | -1.359 | -0.278 | -0.639 | -0.819 |
| 95 | -3.061 | -1.340 | 3.265 | 0.326 | -1.413 | -1.426 | -0.543 | -0.838 | -0.985 |
| 96 | -3.428 | -1.472 | 3.596 | 4.537 | 0.177 | -1.236 | 2.357 | 1.159 | 0.560 |
| 97 | -3.270 | -1.906 | 0.756 | -2.106 | -2.445 | -2.187 | -2.276 | -2.246 | -2.231 |
| 98 | -2.821 | -1.331 | 0.254 | 0.503 | -1.617 | -1.168 | -0.557 | -0.761 | -0.863 |
| 99 | -2.278 | -1.060 | 2.613 | -0.122 | -3.668 | -2.024 | -1.895 | -1.938 | -1.960 |
| 100 | -2.002 | -0.045 | 3.401 | 2.989 | -0.679 | -1.182 | 1.155 | 0.376 | -0.014 |
| 101 | -3.011 | -1.639 | 1.467 | 1.399 | -3.682 | -2.133 | -1.141 | -1.472 | -1.637 |
| 102 | -2.445 | -1.743 | 4.542 | 3.315 | -1.359 | -1.752 | 0.978 | 0.068 | -0.387 |
| 103 | -2.228 | -1.128 | 2.826 | 4.021 | -0.937 | -1.752 | 1.542 | 0.444 | -0.105 |
| 104 | -1.621 | 0.593 | 4.655 | 4.048 | 1.060 | -1.685 | 2.554 | 1.141 | 0.435 |
| 105 | -2.572 | -2.921 | 3.641 | -1.005 | -4.198 | -2.486 | -2.602 | -2.563 | -2.544 |
| 106 | -2.513 | -3.306 | 4.098 | -1.250 | -4.198 | -1.929 | -2.724 | -2.459 | -2.326 |
| 107 | -2.740 | -3.206 | 4.211 | -1.250 | -4.116 | -1.359 | -2.683 | -2.242 | -2.021 |
| 108 | -2.201 | -2.328 | 3.786 | -1.562 | -4.048 | -2.160 | -2.805 | -2.590 | -2.483 |
| 109 | -2.957 | -2.146 | 1.608 | -1.128 | -3.043 | -2.500 | -2.085 | -2.223 | -2.293 |
| 110 | -4.171 | -2.654 | 1.300 | 0.367 | -3.831 | -2.309 | -1.732 | -1.925 | -2.021 |
| 111 | -1.843 | -2.599 | 3.940 | 1.141 | -2.323 | -2.051 | -0.591 | -1.078 | -1.321 |
| 112 | -3.881 | -2.957 | 3.365 | 1.834 | -2.432 | -2.228 | -0.299 | -0.942 | -1.263 |
| 113 | -4.619 | -1.481 | 2.015 | 1.589 | -2.676 | -1.712 | -0.543 | -0.933 | -1.128 |
| 114 | -3.147 | -3.903 | 4.669 | -0.340 | -2.948 | -1.549 | -1.644 | -1.612 | -1.596 |
| 115 | -2.953 | -2.237 | 4.691 | -0.041 | -2.473 | -1.508 | -1.257 | -1.340 | -1.382 |
| 116 | -2.459 | -1.897 | 5.366 | 1.970 | -3.736 | -2.350 | -0.883 | -1.372 | -1.617 |
| 117 | -4.334 | -1.793 | 5.158 | 6.331 | -3.057 | -0.421 | 1.637 | 0.951 | 0.608 |
| 118 | -1.073 | -2.608 | 3.088 | -2.106 | -3.260 | -2.174 | -2.683 | -2.513 | -2.428 |
| 119 | -1.540 | -1.512 | 2.984 | 1.916 | -2.133 | -2.228 | -0.109 | -0.815 | -1.168 |
| 120 | -1.585 | -0.620 | 3.247 | -0.557 | -2.269 | -1.848 | -1.413 | -1.558 | -1.630 |
| 121 | -1.653 | -1.064 | 3.772 | 2.133 | -2.255 | -1.752 | -0.061 | -0.625 | -0.907 |
| 122 | -2.242 | -2.160 | 3.903 | 0.326 | -3.587 | -2.065 | -1.630 | -1.775 | -1.848 |
| 123 | -2.699 | -2.273 | 1.096 | -0.408 | -3.627 | -1.929 | -2.017 | -1.988 | -1.973 |
| 124 | -3.102 | -2.808 | 5.081 | 0.978 | -3.505 | -1.875 | -1.263 | -1.467 | -1.569 |
| 125 | -2.780 | -1.014 | 7.214 | 2.473 | -4.537 | -1.793 | -1.032 | -1.286 | -1.413 |
| 126 | -0.765 | -3.197 | 4.705 | -1.318 | -2.065 | -1.698 | -1.691 | -1.694 | -1.695 |
| 127 | -0.353 | -2.069 | 7.997 | -0.543 | -1.983 | -1.019 | -1.263 | -1.182 | -1.141 |
| 128 | -1.141 | -1.001 | 5.416 | 1.576 | -1.983 | -2.418 | -0.204 | -0.942 | -1.311 |
| 129 | -1.902 | -2.029 | 6.054 | 1.372 | -3.817 | -1.780 | -1.223 | -1.408 | -1.501 |
| 130 | -1.870 | -1.603 | 4.470 | 1.671 | -4.103 | -1.861 | -1.216 | -1.431 | -1.539 |
| 131 | -2.110 | -0.648 | 3.061 | 0.068 | -1.535 | -1.440 | -0.734 | -0.969 | -1.087 |
| 132 | -3.202 | -2.192 | 2.921 | 2.581 | -1.766 | -1.671 | 0.408 | -0.285 | -0.632 |
| 133 | -1.413 | -2.006 | 6.181 | 3.519 | -2.024 | -1.657 | 0.747 | -0.054 | -0.455 |
| 134 | -2.821 | -2.192 | 4.791 | 3.750 | -1.644 | -0.027 | 1.053 | 0.693 | 0.513 |
| 135 | -3.587 | -1.268 | 3.994 | 3.668 | -2.024 | -1.508 | 0.822 | 0.045 | -0.343 |
| 136 | -3.473 | -0.598 | 4.791 | 4.986 | -1.304 | -1.671 | 1.841 | 0.670 | 0.085 |
| 137 | -1.327 | -0.539 | 5.113 | 1.168 | -2.636 | -1.617 | -0.734 | -1.028 | -1.175 |
| 138 | -1.186 | -0.426 | 5.353 | 0.842 | -2.880 | -2.106 | -1.019 | -1.381 | -1.562 |
| 139 | -1.494 | 0.312 | 7.481 | 3.301 | -1.603 | -0.910 | 0.849 | 0.263 | -0.031 |
| 140 | -0.435 | 0.177 | 6.240 | 3.057 | -1.752 | -1.603 | 0.652 | -0.100 | -0.475 |
| 141 | -2.604 | -1.105 | 4.325 | 2.174 | -2.663 | -2.051 | -0.245 | -0.847 | -1.148 |
| 142 | -1.313 | -0.589 | 5.674 | 3.356 | -2.377 | -0.666 | 0.489 | 0.104 | -0.088 |
| 143 | -1.241 | -0.091 | 7.485 | 6.005 | -2.051 | -1.413 | 1.977 | 0.847 | 0.282 |
| 144 | -1.911 | -0.897 | 4.433 | 3.356 | 0.014 | -1.970 | 1.685 | 0.466 | -0.143 |
| 145 | -0.820 | 0.136 | 6.634 | 5.339 | 1.426 | -1.617 | 3.383 | 1.716 | 0.883 |
| 146 | -1.807 | -0.254 | 4.524 | 2.608 | -1.426 | -1.291 | 0.591 | -0.036 | -0.350 |
| 147 | -3.342 | -1.010 | 4.854 | 5.747 | -1.005 | -0.747 | 2.371 | 1.331 | 0.812 |
| 148 | -1.331 | 0.729 | 5.520 | 4.361 | -1.250 | -1.440 | 1.556 | 0.557 | 0.058 |
| 149 | -2.160 | -0.118 | 5.733 | 3.247 | -1.128 | -1.888 | 1.060 | 0.077 | -0.414 |
| 150 | -1.300 | 1.689 | 5.131 | 7.390 | 1.359 | -1.685 | 4.374 | 2.355 | 1.345 |
| 151 | -3.152 | -1.336 | 3.143 | 4.592 | 0.652 | -0.530 | 2.622 | 1.571 | 1.046 |
| 152 | -2.233 | -0.516 | 7.916 | 5.380 | 3.356 | -0.734 | 4.368 | 2.667 | 1.817 |
| 153 | 0.602 | 0.634 | 5.873 | 3.165 | -1.386 | -2.024 | 0.890 | -0.082 | -0.567 |
| 154 | 0.358 | 2.219 | 7.839 | 5.937 | -0.245 | -1.671 | 2.846 | 1.340 | 0.588 |
| 155 | 0.149 | 1.472 | 7.766 | 4.700 | 0.272 | -1.440 | 2.486 | 1.177 | 0.523 |
| 156 | -0.208 | -2.400 | 8.500 | 0.245 | -2.187 | -1.060 | -0.971 | -1.001 | -1.015 |
| 157 | -0.471 | -0.901 | 7.046 | 1.155 | -1.902 | -2.106 | -0.374 | -0.951 | -1.240 |
| 158 | -0.299 | -0.955 | 5.964 | 2.391 | -1.032 | -2.405 | 0.679 | -0.349 | -0.863 |
| 159 | 0.263 | -0.525 | 6.933 | 0.598 | -1.454 | -2.051 | -0.428 | -0.969 | -1.240 |
| 160 | -1.372 | -0.557 | 7.110 | 2.418 | -2.337 | -2.377 | 0.041 | -0.765 | -1.168 |
| 161 | -0.946 | 0.122 | 6.308 | 2.921 | -1.100 | -1.861 | 0.910 | -0.014 | -0.475 |
| 162 | -0.245 | -1.906 | 8.129 | 1.739 | -2.581 | -2.011 | -0.421 | -0.951 | -1.216 |
| 163 | -1.617 | -1.485 | 7.300 | 5.149 | -2.880 | -2.255 | 1.134 | 0.005 | -0.560 |
| 164 | -2.192 | -1.377 | 4.737 | 4.782 | -2.540 | -1.603 | 1.121 | 0.213 | -0.241 |
| 165 | -1.766 | -0.965 | 8.246 | 5.814 | -1.114 | -1.807 | 2.350 | 0.965 | 0.272 |
| 166 | -1.408 | -0.249 | 9.306 | 5.760 | -1.440 | -0.326 | 2.160 | 1.331 | 0.917 |
| 167 | 0.811 | -0.158 | 4.818 | 1.318 | -1.481 | -1.807 | -0.082 | -0.657 | -0.944 |
| 168 | 0.489 | 0.453 | 6.218 | 5.312 | -0.802 | -1.671 | 2.255 | 0.946 | 0.292 |
| 169 | 2.848 | 0.367 | 11.403 | 0.380 | -0.720 | -1.522 | -0.170 | -0.620 | -0.846 |
| 170 | 1.304 | 0.408 | 9.315 | 3.274 | -1.399 | -1.888 | 0.937 | -0.005 | -0.475 |
| 171 | 0.684 | 0.761 | 7.101 | 3.913 | -0.231 | -0.869 | 1.841 | 0.937 | 0.486 |
| 172 | 0.140 | 0.045 | 10.148 | 3.165 | -2.079 | -1.698 | 0.543 | -0.204 | -0.577 |
| 173 | -0.534 | 1.304 | 8.858 | 3.885 | -1.277 | 0.353 | 1.304 | 0.987 | 0.829 |
| 174 | -0.729 | 0.326 | 8.785 | 7.513 | -0.516 | -1.549 | 3.498 | 1.816 | 0.975 |
| 175 | 0.376 | 0.711 | 9.963 | 4.619 | -0.530 | -1.617 | 2.045 | 0.824 | 0.214 |
| 176 | -0.457 | 1.436 | 8.169 | 7.173 | 2.214 | -1.589 | 4.694 | 2.599 | 1.552 |
| 177 | -0.747 | 2.146 | 9.582 | 7.567 | 1.888 | -1.562 | 4.728 | 2.631 | 1.583 |
| 178 | -0.512 | 0.023 | 9.569 | 7.458 | 0.706 | -1.494 | 4.082 | 2.223 | 1.294 |
| 179 | -1.644 | 0.045 | 9.347 | 7.893 | 3.994 | -0.258 | 5.944 | 3.876 | 2.843 |
| 180 | 1.988 | 0.444 | 7.073 | 4.130 | -1.182 | -2.092 | 1.474 | 0.285 | -0.309 |
| 181 | -0.399 | 2.436 | 9.677 | 6.535 | 0.421 | -0.720 | 3.478 | 2.079 | 1.379 |
| 182 | 1.196 | 3.079 | 13.024 | 6.222 | -0.122 | -1.304 | 3.050 | 1.599 | 0.873 |
| 183 | 1.612 | 2.436 | 9.360 | 7.078 | -0.842 | -1.916 | 3.118 | 1.440 | 0.601 |
| 184 | 1.245 | 3.990 | 11.398 | 10.352 | 2.636 | -0.869 | 6.494 | 4.039 | 2.812 |
| 185 | -0.394 | -1.005 | 9.854 | 7.676 | 1.807 | -1.549 | 4.741 | 2.645 | 1.596 |
| 186 | 0.838 | 3.256 | 10.814 | 9.469 | 3.627 | -0.095 | 6.548 | 4.334 | 3.226 |
| 187 | -0.969 | 1.177 | 7.422 | 5.461 | 0.489 | -1.943 | 2.975 | 1.336 | 0.516 |
| 188 | -0.725 | 2.631 | 11.380 | 11.384 | 2.309 | -0.584 | 6.847 | 4.370 | 3.131 |
| 189 | 3.048 | 0.783 | 11.498 | 1.861 | -0.666 | -1.997 | 0.598 | -0.267 | -0.700 |
| 190 | 1.476 | 0.466 | 12.050 | 5.217 | -1.141 | -2.350 | 2.038 | 0.575 | -0.156 |
| 191 | 1.934 | 1.150 | 8.215 | 3.546 | -0.639 | -1.902 | 1.454 | 0.335 | -0.224 |
| 192 | 1.499 | 3.455 | 13.069 | 5.706 | 0.747 | -1.807 | 3.226 | 1.549 | 0.710 |
| 193 | 0.195 | 0.611 | 11.720 | 6.181 | -0.516 | -1.766 | 2.833 | 1.300 | 0.533 |
| 194 | 3.935 | 2.808 | 14.803 | 6.521 | 0.163 | -1.671 | 3.342 | 1.671 | 0.835 |
| 195 | 2.341 | 3.233 | 13.264 | 7.648 | 0.625 | -0.883 | 4.137 | 2.463 | 1.627 |
| 196 | 3.387 | 2.559 | 15.030 | 7.621 | 0.217 | -1.535 | 3.919 | 2.101 | 1.192 |
| 197 | 3.442 | 4.614 | 15.288 | 5.855 | 1.685 | -0.897 | 3.770 | 2.214 | 1.437 |
| 198 | 2.531 | 2.663 | 15.084 | 8.708 | 1.196 | -1.345 | 4.952 | 2.853 | 1.803 |
| 199 | 1.481 | 1.105 | 13.572 | 9.469 | 0.802 | -1.576 | 5.135 | 2.898 | 1.780 |
| 200 | 0.792 | 3.066 | 14.459 | 10.678 | 3.165 | -0.625 | 6.922 | 4.406 | 3.148 |
| 201 | 3.251 | 3.383 | 13.164 | 11.520 | 3.002 | -0.272 | 7.261 | 4.750 | 3.495 |
| 202 | 1.730 | 0.562 | 14.790 | 13.612 | 5.353 | 0.516 | 9.483 | 6.494 | 4.999 |
| 203 | 3.469 | 4.202 | 12.172 | 7.377 | -0.394 | -1.861 | 3.491 | 1.707 | 0.815 |
| 204 | 2.559 | 5.461 | 16.334 | 12.335 | 3.858 | -0.543 | 8.097 | 5.217 | 3.777 |
| 205 | 3.519 | 3.274 | 15.465 | 4.904 | 0.543 | -1.766 | 2.724 | 1.227 | 0.479 |
| 206 | 5.679 | 5.271 | 19.060 | 7.934 | -0.095 | -1.291 | 3.919 | 2.183 | 1.314 |
| 207 | 5.303 | 6.489 | 22.429 | 12.227 | 1.508 | -0.611 | 6.867 | 4.374 | 3.128 |
| 208 | 3.894 | 3.102 | 19.807 | 13.341 | 4.877 | 0.408 | 9.109 | 6.208 | 4.758 |
| 209 | 8.301 | 10.384 | 23.838 | 15.800 | 4.116 | 0.978 | 9.958 | 6.965 | 5.468 |
